# Comprehensive Proteomic Analysis of Spider Dragline Silk from Black Widows: A Recipe to Build Synthetic Silk Fibers

**DOI:** 10.3390/ijms17091537

**Published:** 2016-09-13

**Authors:** Camille Larracas, Ryan Hekman, Simmone Dyrness, Alisa Arata, Caroline Williams, Taylor Crawford, Craig A. Vierra

**Affiliations:** Department of Biological Sciences, University of the Pacific, Stockton, CA 95211, USA; c_larracas@u.pacific.edu (C.L.); r_hekman@u.pacific.edu (R.H.); s_dyrness1@u.pacific.edu (S.D.); aarata1@pacific.edu (A.A.); c_williams15@u.pacific.edu (C.W.); taylor.rabara@gmail.com (T.C.)

**Keywords:** dragline silk, major ampullate, proteomics, black widow spider, cob-weaver, spidroin

## Abstract

The outstanding material properties of spider dragline silk fibers have been attributed to two spidroins, major ampullate spidroins 1 and 2 (MaSp1 and MaSp2). Although dragline silk fibers have been treated with different chemical solvents to elucidate the relationship between protein structure and fiber mechanics, there has not been a comprehensive proteomic analysis of the major ampullate (MA) gland, its spinning dope, and dragline silk using a wide range of chaotropic agents, inorganic salts, and fluorinated alcohols to elucidate their complete molecular constituents. In these studies, we perform in-solution tryptic digestions of solubilized MA glands, spinning dope and dragline silk fibers using five different solvents, followed by nano liquid chromatography coupled to tandem mass spectrometry (LC-MS/MS) analysis with an Orbitrap Fusion™ Tribrid™. To improve protein identification, we employed three different tryptic peptide fragmentation modes, which included collision-induced dissociation (CID), electron transfer dissociation (ETD), and high energy collision dissociation (HCD) to discover proteins involved in the silk assembly pathway and silk fiber. In addition to MaSp1 and MaSp2, we confirmed the presence of a third spidroin, aciniform spidroin 1 (AcSp1), widely recognized as the major constituent of wrapping silk, as a product of dragline silk. Our findings also reveal that MA glands, spinning dope, and dragline silk contain at least seven common proteins: three members of the Cysteine-Rich Protein Family (CRP1, CRP2 and CRP4), cysteine-rich secretory protein 3 (CRISP3), fasciclin and two uncharacterized proteins. In summary, this study provides a proteomic blueprint to construct synthetic silk fibers that most closely mimic natural fibers.

## 1. Introduction

Spider dragline silk has exceptional mechanical properties, including high tensile strength and toughness [1]. Dragline silk from *Nephila clavipes* has been reported as a two-protein fiber, consisting of the major ampullate spidroins 1 and 2 (MaSp1 and MaSp2) [2,3]. Since this discovery, labs across the world have focused on the production and purification of recombinant MaSp1 and MaSp2, coupled with the development of wet-spinning methodologies to synthesize artificial silk fibers [4,5,6,7,8]. However, recent biochemical evidence is emerging that supports additional proteins are spun into dragline silk. In fact, a new paradigm is surfacing that suggests dragline silk consists of additional spidroin family members as well as low molecular weight proteins that are spun with MaSp1 and MaSp2 [9,10]. With advances in technology, there is a pressing need to develop new biomaterials that have outstanding material properties and are environmentally friendly. Therefore, in order to produce high performance synthetic spider silk, one of the major hurdles involves a comprehensive understanding of the proteins spun into the fibers, as well as proteins that participate in the assembly process. Another barrier that must be circumvented involves production of large quantities of recombinant silk proteins in a cost-efficient manner and mastery of an artificial spinning process that mimics the natural process.

Attempts to elucidate the molecular components that comprise dragline silk have involved profiling the amino acid composition of threads, screening cDNA libraries, and performing transcriptomic and proteomic analyses of the silk-producing glands and fibers [9,11,12,13,14,15,16]. Much insight into the mechanism of silk extrusion as well as protein composition of dragline silk fibers has come from the orb-weaver *N. clavipes* [8,13,17,18,19]. However, one spider species that is rapidly emerging as a prominent model system to elucidate the molecular constituents of the silk assembly pathway and protein-fiber composition is *Latrodectus hesperus*, a cob-weaver spider that belongs in the family Theridiidae. Increasing numbers of transcriptome and proteome studies, along with on-going work on its genome, is revealing much insight into spider silk biology. Expression studies that utilize massively parallel signature sequencing (MPSS) to profile mRNA transcript levels from the major ampullate (MA) glands of *L. hesperus* have identified genes that are highly transcribed by specialized epithelial cells that reside in the tail region of the gland, leading to a list of candidate mRNAs that are potentially translated, secreted, and transported into the luminal contents of the ampulla, ultimately being integrated into the final silk product [11]. Although these transcriptomic studies provide strong candidate genes coding for potential products involved in the silk assembly process or fibers, further confirmation at the protein level is necessary to determine which products are secreted into the spinning dope and subsequently extruded into fibers.

Limited numbers of proteomic studies using mass spectrometry have been performed on dragline silk and silk-producing glands. One study has led to the identification of a low molecular cysteine-rich protein 1 (CRP1) as a component of dragline silk [10]. In addition, a recent proteomic study has suggested that aciniform spidroin 1 (AcSp1) is a constituent of dragline silk, which has been further supported by its presence in the MA gland [9]. These findings suggest that spiders alter the mechanical properties of their fibers by changing silk gene expression and silk protein composition. In our studies, we employed a wide range of solvents that have different chemical and physical properties to dissolve dragline silk, its spinning dope, and the MA gland, followed by comprehensive proteomic analyses using the products from in-solution tryptic digestions to improve our understanding of the mechanism of silk assembly and fiber composition. Collectively, our studies are designed to help advance the silk community’s understanding of the protein constituents present within dragline silk. These findings have implications for scientists that are interested in large-scale production of synthetic dragline silk from recombinant protein sources, as they provide a basic recipe for artificial fiber production.

## 2. Results

### 2.1. Diverse Chemical Solvent Treatments of MA Glands, Its Spinning Dope, and Dragline Silk Fibers Reveal Differential Protein Solubilization

**Figure 1:**
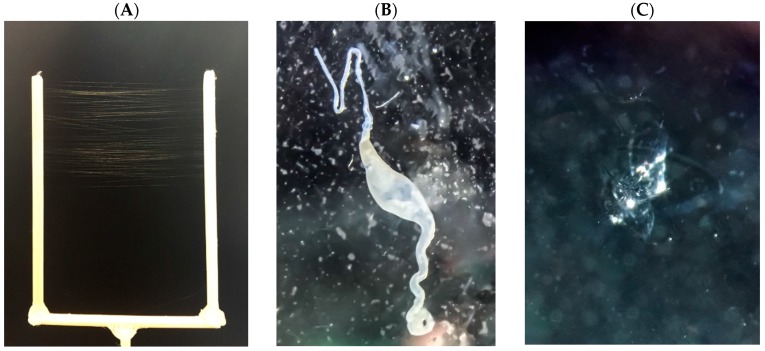
Isolation of dragline silk and major ampullage (MA) gland from *L. hesperus*: (**A**) digital photograph of dragline silk wrapped on a Y-apparatus; (**B**) light microscope image of a single MA gland shown at 10× magnification; and (**C**) clear viscous spinning dope (center).

To date, a direct comparison of the efficiency of different solvents and their ability to dissolve MA constituents has not been reported. In order to identify new protein constituents of dragline silk, we isolated fibers from female black widow spiders (Figure 1A). Individuals were allowed to “freefall” and dragline silk was wrapped onto a Y-apparatus for biochemical analysis. Dragline silk was treated with a broad range of solvents with different physical and chemical properties to investigate solubilization efficiencies. Treatments included chaotropic agents, alcohols and salts. The MA gland and spinning dope was also used for the analysis (Figure 1B,C).

**Figure 2:**
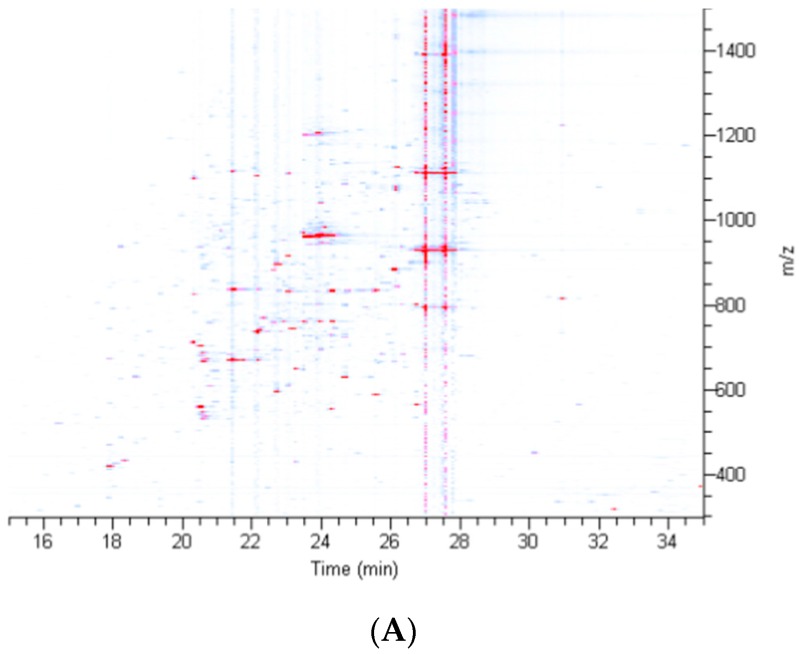

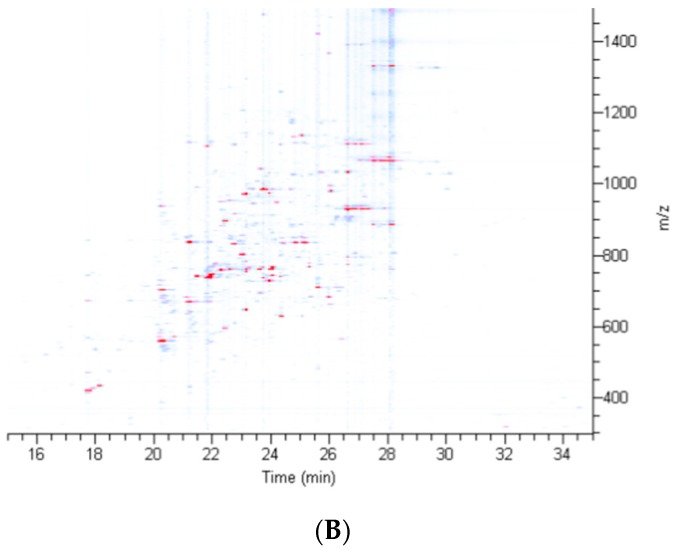
Density map of in-solution tryptic digestion products obtained from dragline silk treated with different chemical solvents: (**A**) hexafluoroisopropanol (HFIP); and (**B**) urea. The *X*-axis reveals the retention time (min) for the precursor ions eluted during nano-LC MS analysis, while the *Y*-axis shows the *m*/*z* values (*z* = +2 to +8). The *Z*-axis (color) is intensity, normalized to 2.0 × 10^7^ arbitrary units.

After sufficient quantities of silk were collected, equivalent amounts of dragline silk were treated with five different solvents: guanidine thiocyanate (GITC), lithium bromide (LiBr), guanidine hydrochloride (GdnHCl), hexafluoroisopropanol (HFIP) and urea. These solvents were selected because they display a broad range of chemical properties that facilitate protein denaturation and solubilization and are commonly used for silk and tandem mass spectrometry (MS/MS) analysis. GITC treatment led to immediate solubilization of dragline silk, while HFIP and LiBr, although slower in solubilization, were able to completely dissolve the fibers within 30 min. Both GdnHCl and urea were unable to fully dissolve the fibers even after 3 h of treatment [20]. Solubilized proteins were then subject to in-solution tryptic digestion and products were examined using nano liquid chromatography coupled to tandem mass spectrometry (LC-MS/MS) analysis coupled to an Orbitrap Fusion™ Tribrid™ mass spectrometer. Density maps generated from the proteomic data using the different solvent systems revealed complex tryptic peptide precursor ion patterns (Figure 2 and Figure S1). The majority of the peptide precursor ions eluted from the C18 resin 18–28 min into the chromatographic run, displaying mass to charge (*m/z*) values (*z* = +2 to +8) that spanned 600 to 1100 (Figure S1). Consistent with the qualitative observations, GITC, LiBr, and HFIP treatment led to the most efficient solubilization and largest number of precursor peptide ions, while urea and GdnHCl were not as effective (Figure 2A,B and Figure S1).

### 2.2. Treatment of Dragline Silk with GITC Leads to the Identification of the Highest Number of Proteins by MS/MS Analysis

**Figure 3:**
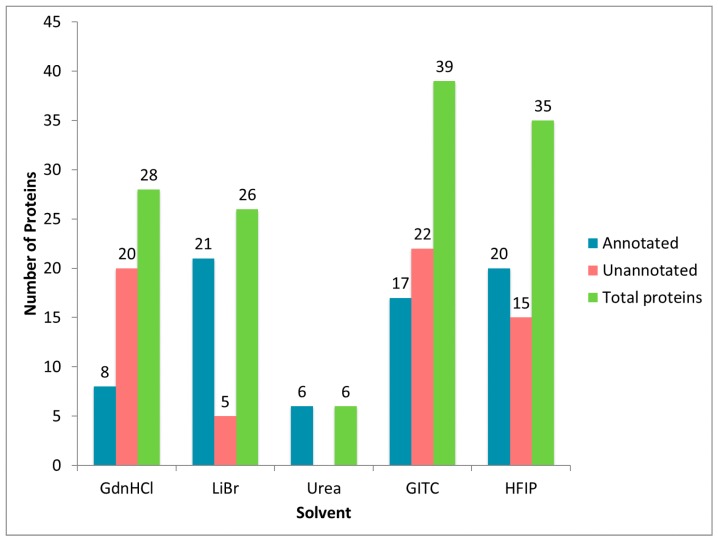
Total number of unique proteins identified by Proteome Discoverer 2.1 after searching ion fragment spectra against UniProt and transcriptome databases using dragline silk dissolved in different solvents, followed by in-solution tryptic digestion. Note: The UniProt database contains several unique accession numbers for MaSp1 and MaSp2 variants.

In order to obtain a comprehensive perspective of the total number of proteins found in dragline silk, the MA gland, and the spinning dope, we performed MS/MS analysis of the tryptic peptides derived from solubilization with GITC, LiBr, GdnHCl, HFIP and urea. In order to further maximize protein identification, we performed MS/MS analysis using three different dissociation modes for peptide precursor ions: collision-induced dissociation (CID), electron transfer dissociation (ETD), and high energy collision dissociation (HCD) by the Orbitrap Fusion™ Tribrid™ mass spectrometer. For each chemical treatment, spectra were combined (all three dissociation modes) and first analyzed using Proteome Discoverer 2.1 against the UniProt *Latrodectus* database (annotated). Spectra that failed to identify annotated proteins in the UniProt database were then searched against the *Latrodectus* transcriptome database (unannotated). Computational analyses revealed that dragline silk solubilized with GITC yielded 39 protein accession numbers, which was the highest number. HFIP treatment identified the second highest protein number at 35 (Figure 3). Treatment with GdnHCl and LiBr led to the discovery of 28 and 26 proteins, respectively. Urea-treated dragline silk was the least efficient at solubilization and protein identification, yielding six proteins (Figure 3). Because sequences for MaSp1 and MaSp2 have multiple protein accession numbers in the UniProt database (e.g., MaSp1 loci 1, 2, and 3), we compressed these spidroin identifications down to either MaSp1 or MaSp2, eliminating the potential for duplications based on different accession numbers (Figure 3). After elimination of all redundancies from the dataset, a composite list of all of the proteins identified in dragline silk for all solvents revealed 28 unique proteins. Twenty had annotation and eight represented unannotated, uncharacterized proteins (Figure S2).

### 2.3. Different Solvent Systems Solubilize Dragline Silk Proteins with Different Efficiencies

**Figure 4:**
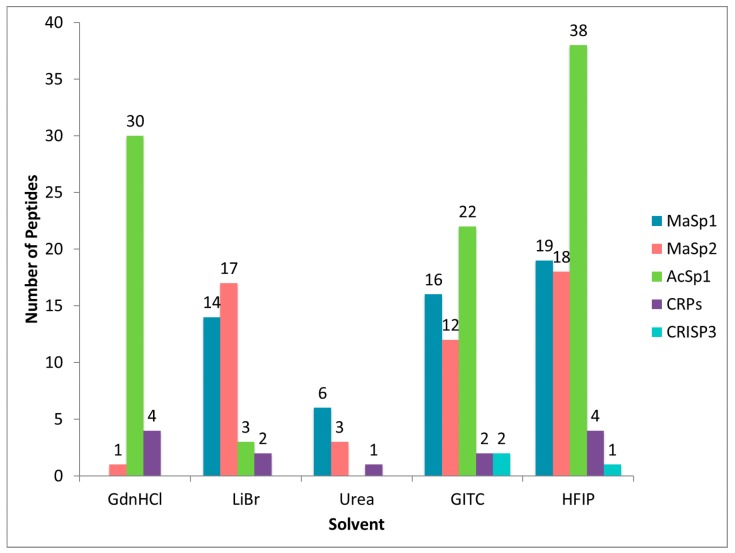
Total number of peptides identified that match either of the major ampullate spidroins (MaSp1 and MaSp2), aciniform spidroin 1 (AcSp1), any of the cysteine-rich protein family members (CRPs), or cysteine-rich secretory protein 3 (CRISP3) from dragline silk.

Of the 28 unique proteins identified in dragline silk, several were repeatedly identified across different solvent treatments. However, it became quite evident that despite using dragline silk samples collected at the same time from one individual, the solubility of these proteins varied based upon solvent choice (Figure S2). Consistent with this assertion is the observation that MaSps were highly represented when dragline silk was treated with LiBr, GITC, and HFIP, giving rise to similar numbers of tryptic peptides derived from these fibroins (Figure 4). However, the MaSps were not as efficiently dissolved in urea and GdnHCl, an observation supported by the detection of fewer peptides by the MS/MS analysis. Consistent with previous reports, we also identified AcSp1 in dragline silk [9]. Treatment with GdnHCl, GITC, and HFIP revealed large numbers of peptide matches for AcSp1; the number of peptide matches ranged from 0–38 (Figure 4). In addition, we identified peptides corresponding to three members belonging to the low molecular weight cysteine-rich protein family (CRPs) and another, uncharacterized cysteine-rich protein named cysteine-rich secretory protein 3 (CRISP3). Peptides derived from CRISP3 were identified from HFIP and GITC-treatments, while CRP family members were detected in every tested solvent (Figure 4). In summary, HFIP-treated dragline silk led to highest number of peptides covering the MaSps, AcSp1, CRPs and CRISP3, which was followed in efficiency by GITC treatment. Urea treatment yielded the lowest number of peptides corresponding to these major protein constituents (Figure 4).

### 2.4. CRP1, CRP2 and CRP4 Are Present within MA Glands, Spinning Dope, and Dragline Silk Fibers

**Figure 5:**
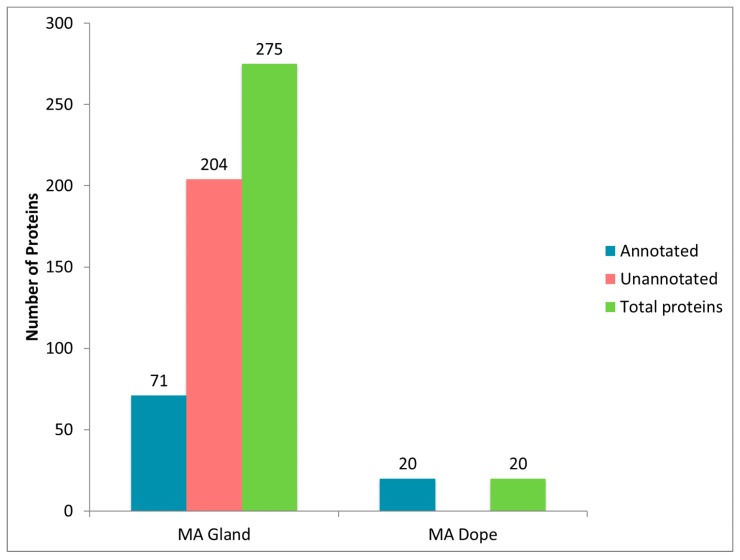
Identification of unique proteins within the major ampullate (MA) gland and spinning dope of the black widow spider.

Because HFIP was one of the most effective solvents at dissolving dragline silk and it generated robust protein identification, we investigated protein composition of the MA glands and its spinning dope after HFIP treatment. For these studies, a single female black widow spider was dissected to remove both MA glands (two are present in one individual), dissolving one in HFIP, while the other was used to isolate its spinning dope. The advantage of testing the spinning dope is that it exclusively represents fluid stored in the ampulla and lacks the epithelial cells that are present within the tail region of the MA gland. Although the epithelial cells in the tail region represent the major site of synthesis for fiber materials, these cells also contain proteins involved in metabolism, protein synthesis and other basic cellular activities. As anticipated, the total number of proteins identified with the MA gland surpassed the total number of proteins present within the spinning dope (Figure 5). Seventy-one unique protein accession numbers (annotated) were identified from the HFIP-treated MA gland after tryptic digestion and MS/MS analysis, while 20 unique protein accession numbers were identified in the spinning dope (Figure 5). Many of the proteins identified in the MA gland could be classified as proteins involved in protein folding, protein synthesis, energy production, or spidroin family members (Figure S3). Many unannotated proteins in the MA gland likely fall under the classification of factors involved in cellular metabolism.

**Figure 6:**
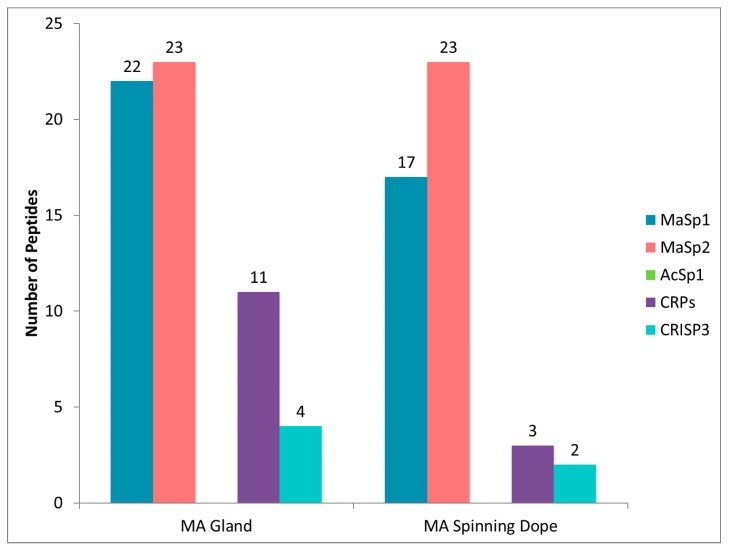
Number of distinct peptides identified by tandem mass spectrometry analysis after hexafluoroisopropanol treatment of the major ampullate (MA) gland and its spinning dope, followed by in-solution tryptic digestion. Corresponding proteins are indicated by different colors.

Further analysis of the annotated proteins revealed multiple, distinct accession numbers corresponding to MaSp1 and MaSp2, which we compressed down to single protein counts. After this modification, 66 distinct proteins were present within the gland, and 11 in the dope. As previously mentioned, because dragline silk fibers are derived from the spinning dope during extrusion, we hypothesized that this liquid contains the main protein constituents that are spun into fibers. Based upon this assertion, we focused our attention on the 11 proteins that had unique protein accession numbers identified within the spinning dope. All proteins in the spinning dope were determined to have annotation. These included MaSp1, MaSp2, CRPs (CRP1, CRP2 and CRP4), CRISP3, fasciclin, actin, α-2-macroglobulin, and two uncharacterized proteins (G6CHZ1, and E7D1R8). Large numbers of distinct peptides could be found corresponding to regions within the primary sequence of MaSp1 and MaSp2, an observation consistent with the glandular function of the MA and MaSp synthesis and storage (Figure 6). Albeit lower numbers of peptides, both the CRPs and CRISP3 proteins were present in the MA gland and dope (Figure 6). Lower peptide numbers for the CRPs and CRISP3 were clearly attributed to the fact that these molecules have shorter protein chain lengths (<10 kDa). Peptides corresponding to AcSp1 were not identified in the MA gland or spinning dope.

**Figure 7:**
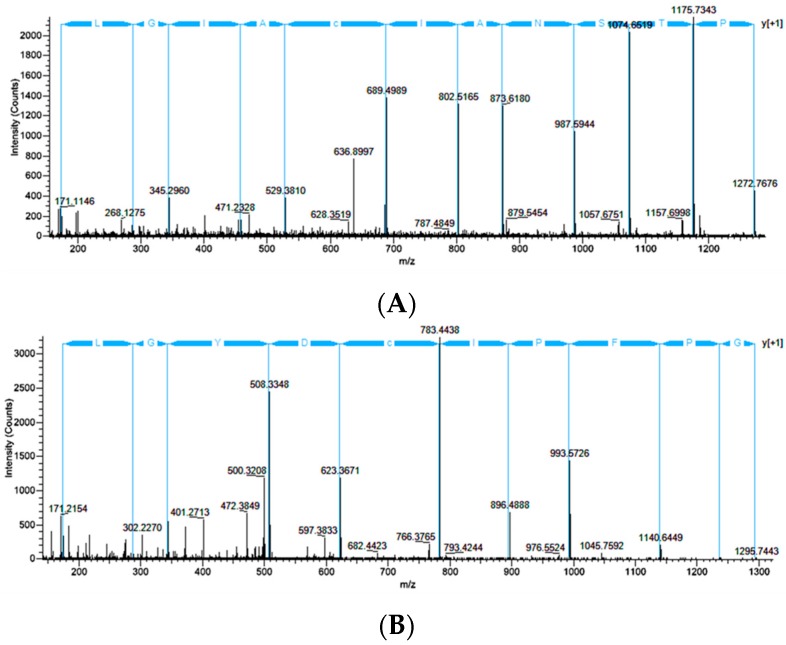

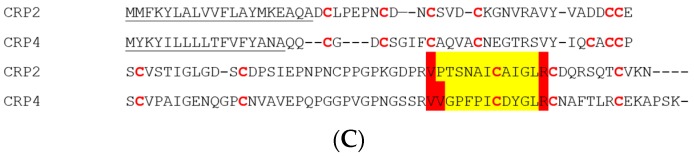
Ion fragment spectra for cysteine-rich proteins 2 and 4 (CRP2 and CRP4) after treatment of dragline silk with hexafluoroisopropanol and tryptic digestion, along with their primary sequences: (**A**) High energy collision dissociation (HCD) spectrum of precursor ion with MH^+^ = 1371.73 (*z* = +2) derived from CRP2 with y-ion fragmentation pattern above in blue; (**B**) HCD spectrum of precursor ion with MH^+^ = 1492.75 (*z* = +3) generated from CRP4 with y-ion fragmentation pattern above in blue; and (**C**) the yellow block regions correspond to the peptide segment identified by de novo sequencing. Red blocked regions did not yield strong y or b-ions. Underlined regions represent putative signal secretion signals determined by SignaIP 4.1 [21]. Bold letters for Cys (**C**) are conserved residues found in CRP family members and dashes are insertions for alignment purposes.

Previous studies involving screening cDNA libraries from black widow spiders have revealed at least five distinct CRP family members (CRP1-5), although their exact functional significance and expression patterns in silk-producing glands or fibers have not been firmly established [10]. Because the CRPs have conserved regions within their primary sequences, we searched our MS/MS spectra to identify peptide regions that could distinguish individual CRP family members from one another. Consistent with previous findings, we identified the presence of CRP1 [9,10]. We also confirmed the presence of CRP2 and CRP4 proteins within MA glands, its spinning dope, and dragline silk fibers. MS/MS analysis of peptide precursor ion MH^+^ = 1371.73 (*z* = +2) predicted the amino acid sequence PTSNAICAIGL, which mapped to the C-terminal region of CRP2 (Figure 7A–C). MS/MS analysis of peptide precursor ion MH^+^ = 1492.75 (*z* = +3) was shown to have the peptide sequence VVGPFPICDYGLR, a segment that was unique and also localized to the C-terminus of CRP4 (Figure 7B,C). For both CRP2 and CRP4, which are approximately 90 amino acids in length, we were able to find at least two tryptic peptides from each CRP, which corresponded to about 62% sequence coverage. No peptides derived from CRP3 or CRP5 were identified as constituents of dragline silk, the spinning dope or MA gland. Taken together, these results support the expression of CRP1, CRP2, and CRP4 in the MA gland and their extrusion into dragline silk.

### 2.5. Comparison of Proteomic Data Sets

**Figure 8:**
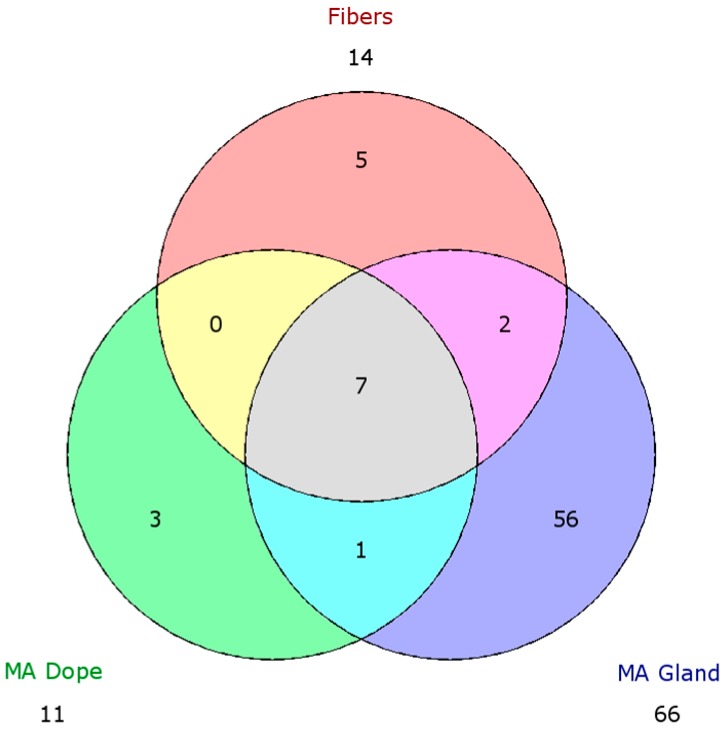
Venn diagram for unique proteins identified from dragline silk, major ampullate (MA) dope, and the MA gland after solubilization in hexafluoroisopropanol and in-solution tryptic digestion, followed by tandem mass spectrometry analysis. The number in the red circle designates the number of unique proteins identified only in fibers, green for the dope, and blue for the gland tissue. Numbers in the overlapping regions (yellow, pink and cyan) designate numbers of unique proteins found in both adjacent categories (e.g., Two in the pink region mean 2 unique proteins were found both in gland tissue and fibers). The central gray region designates unique proteins common between fibers, gland tissue, and spinning dope.

In order to search for the most fundamental ingredients that encompass dragline silk, we generated a Venn diagram using HFIP-identified proteins from the MA gland, spinning dope and dragline silk and looked for an overlapping presence of proteins. Sixty-six distinct proteins were identified from the MA gland, 14 from dragline silk fibers, and 11 from the spinning dope (Figure 8). Fifty-six proteins identified from the MA gland were not present within the fibers or spinning dope. These proteins displayed a diverse range of biological activities, ranging from protein synthesis to metabolism. Three proteins were exclusively found in the spinning dope, one represented a protein with a predicted molecular mass of 24.6 kDa. Over 20% of this protein was comprised of Lys (12.7%) and Asp (8.6%). AcSp1 was found in the MA gland and dragline silk fibers, but were not detected in the spinning dope (Figure 8). In addition, we detected five proteins that were only found in the fibers, which included AcSp1-like, pyriform spidroin (PySp1), cystatin, and two uncharacterized proteins (E7D1H9, and E7D1M7). Of particular interest were the seven proteins identified in the MA gland, fibers, and spinning dope. These proteins included MaSp1, MaSp2, CRP1, CRP2, CRP4, CRISP3, and fasciclin.

**Figure 9:**
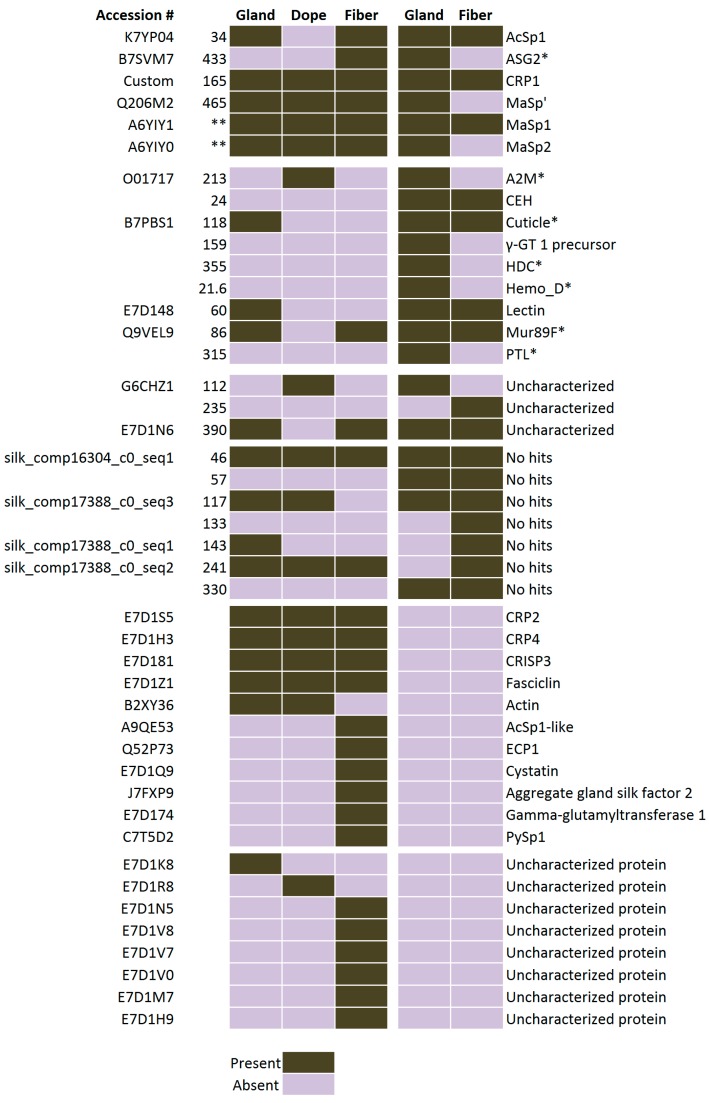
Chart of presence (black) or absence (light purple) of proteins that are predicted within the MA gland, dope and fiber. Two proteomic data sets are available from *L. hesperus* for comparative purposes. The left panel (gland, dope, and fiber) were conducted with an Orbitrap Fusion™ Tribrid™ mass spectrometer with five different solvent treatments and three modes of peptide ion dissociation, CID, HCD, and ETD, while the right panel (gland, and fiber) was conducted using a LTQ Orbitrap Velos™ mass spectrometer and one mode of ion dissociation, CID [9]. Accession numbers, scaffolding IDs, and silk specific transcripts (SSTs) are listed on the left [9]. Custom indicates the sequence was added into the database manually. No hits indicate the sequences do not match anything in the databases. * Indicate some peptide sequences that were derived from SSTs that are uncharacterized in *L. hesperus*, but are thought to be homologous to known proteins in different species [9]; ** Indicate protein types with more than one predicted protein [9].

In order to build a comprehensive profile of the proteins found in black widow dragline silk, we assembled a chart from two distinct proteomic data sets collected from *L. hesperus* (Figure 9). Using our five different solvent treatments, we were able to detect CRP2 in every sample, while CRP4 was detected in GdnHCl and HFIP treated samples, despite a previous study that failed to report their presence in the MA gland or fibers [9].

## 3. Discussion

### 3.1. Fundamental Knowledge of Silk Extrusion Pathway

During the spinning process of dragline silk, current models support that spidroins are stored in the ampulla of the MA gland and are extruded via the spinning duct, which ultimately exits the spigots located on the spinneret. As the spidroins move through the spinning duct, the spidroins experience physical and biochemical changes that promote a liquid–liquid phase separation, followed by a liquid–solid phase transition that produces a preliminary silk fiber [22,23]. After extrusion, the preliminary fiber undergoes a post-spin draw, where the remaining solvent evaporates in the air, leading to the formation of a high performance fiber. Biophysical studies support that the spidroins exist in a liquid crystalline phase, allowing for the spidroins to flow in a pre-aligned fashion that promotes alignment of the protein chains along the axis of the spinning duct, which facilitates the formation of β-sheet crystals [24,25,26]. Little information is known whether other protein components play significant biological roles that facilitate spidroin storage at high concentrations within the ampulla, spidroin assembly, and/or enhancement of mechanical properties.

### 3.2. Proteomic Analysis with Different Chemical Solvents

Although dragline silk has been treated with different solvents to investigate their impact on fiber mechanics and supercontraction properties [27,28], no proteomic studies that encompass a broad range of different solvents have been performed to provide a comprehensive perspective of molecules involved in the silk assembly pathway and fibers. To provide a thorough analysis of the molecular components involved in silk extrusion as well as identify novel constituents of dragline silk, we used five solvents with different chemical properties, along with a powerful mass spectrometer with three different modes of peptide dissociation to identify proteins in the MA gland, its spinning dope, and dragline silk fibers. We demonstrate that certain solvents are better suited for solubilizing specific proteins in the MA gland, the spinning dope, and the fibers. Based upon proteins that have unique accession numbers in the database, three solvents were shown to be the most efficient at dissolving black widow dragline silk proteins on a global scale; these included LiBr, GITC, and HFIP. Urea, which has been commonly used in many studies to dissolve dragline silk, was determined to yield fewer protein identifications by mass spectrometry. However, we did observe that certain proteins were more preferentially dissolved by specific solvents. For example, in our studies we identified the presence of AcSp1 in five out of the five solvents tested, with the most peptides being identified with HFIP, followed by GdnHCl and then GITC. LiBr treatment led to fewer AcSp1 peptides after tryptic digestion, but urea did not efficiently dissolve AcSp1 in dragline silk. In part, this could be one of the primary reasons that AcSp1 has been difficult to detect as a constituent of dragline silk since a large number of sample buffers utilized in the field use urea. Another reason could be that the black widow spiders transiently express this gene in the MA gland, offering cob-weavers the ability to build different composite materials with mechanical properties based upon dietary or environmental conditions. In our proteomic study, we used one individual to collect dragline silk and another individual to collect the MA gland and spinning dope (one spider has two MA glands). We did discover AcSp1 in the fibers with four different solvent treatments, but did not find peptides corresponding to AcSp1 in the MA gland or spinning dope. The most likely explanation for this observation centers on the fact that two different individuals were used: one for the fiber collection and one for the MA glandular materials. This is consistent with the model that different individuals can transiently express AcSp1 in the MA gland, a property likely associated with energy resource availability. Evidence to buttress this assertion is supported by the detection of AcSp1 mRNA and protein in the MA gland from black widow spiders in a transcriptomic and different proteomic study [9,12]. To date, MA fibers have been reported in the scientific literature to display, at times, a large amount of variation with respect to their mechanical properties. Much of this variation has been attributed to humidity, reeling speed during fiber collection, MaSp2 levels, spider species and temperature [29,30,31]. However, it is also interesting to speculate whether differences in mechanical properties are associated, in part, with varying quantities of AcSp1 in the fibers. Given the similarity in the predicted amino acid composition profiles for AcSp1 and MaSps, it would seem plausible that the presence of AcSp1 in dragline silk fibers could have been overlooked by the silk community. Future studies will undoubtedly resolve this issue.

### 3.3. CRP1, CRP2 and CRP4 Are Present within Dragline Silk Fibers

Collectively, our proteomic studies support the presence of seven common proteins that are present within the MA gland, spinning dope, and dragline silk fibers. These proteins consist of MaSp1, MaSp2, CRP1, CRP2, CRP4, CRISP3, and fasciclin. All variants of MaSp1 were found in our studies (e.g., products from MaSp1 locus 1, 2, and 3), which indicate that dragline silk incorporates products derived from all three MaSp1 loci. A recent transcriptome analysis has shown that fasciclin mRNA transcript levels are highly expressed in the MA gland of *L. hesperus*. Interestingly, in this same study, CRISP3, a cysteine-rich secretory protein, was identified in the transcriptome analysis to have higher levels of the antisense mRNA, which might imply CRISP3 could potentially be translationally regulated [11]. AcSp1 represents another component of dragline silk, but its expression pattern appears to be transient in nature, which could explain why differential results have been reported regarding its presence in the MA gland and dragline silk fibers. Although a previous proteomic study did not detect the presence of CRP2 and CRP4 in dragline silk fibers from black widow spiders, the inability to detect these proteins in this study could be due to the following reasons: (1) these studies involved in-gel protease digestions, where samples were excised from SDS-PAGE gels after staining with Coomassie blue and given the low molecular weight of the CRPs, these proteins could have migrated off the gel and excluded from the analysis; (2) these studies used samples that were co-digested with chymotrypsin and trypsin, which provide more cleavage sites, generating shorter peptides that often elude MS/MS analysis because standard parameters set during data-dependent acquisition eliminate sequencing short peptides; (3) these studies applied one type of peptide fragmentation, CID, where our analysis used three types, CID, HCD, and ETD; (4) these studies used a mass spectrometer with lower sensitivity limits (LTQ Orbitrap Velos™ versus Orbitrap Fusion™ Tribrid™); (5) these studies did not employ a wide range of diverse solvents and additionally, samples were processed by in-gel tryptic digestion, which is known to be less efficient than in-solution digestions.

In our studies, we found up to 62% sequence coverage for CRP2 and CRP4, which provides strong evidence for a their presence and supports that they play a central role in dragline silk assembly and/or fiber mechanics. One intriguing question involves the biological function of the CRPs. Future experiments will focus on determining whether the CRPs can form direct physical interactions with the spidroins or other proteins in the spinning dope. Although pH changes have been reported to trigger spidroin aggregation during extrusion, it will be interesting to investigate whether CRP tertiary structures are altered in response to pH changes and whether they participate in the regulation of spidroin assembly and packing process.

### 3.4. New Paradigm Shift Is Emerging that Supports Different Silk Types Are Spun from Multiple Spidroin Family Members

Recent transcriptome studies have detected tubuliform spidroin 1 (TuSp1) transcripts in the MA gland, implying that the TuSp1 protein could be potentially present in dragline silk [11]. In our studies, we were unable to detect the TuSp1 protein in dragline silk, the MA gland, or the spinning dope. This seems to imply that TuSp1 mRNA could be translated at specific times to form composite fibers, similar to AcSp1 in dragline silk. Alternatively, it could suggest that the MA gland in females is capable of shifting synthesis to tubuliform silk during the reproductive stage when egg sacs are spun and require large amounts of tubuliform silk. Given the similar diameter sizes of tubuliform and MA silks (~5 microns), this switch could not be readily distinguished by scanning electron microscopy. Further work will need to be conducted to determine what cues trigger translation of the TuSp1 mRNA. Intriguingly, MaSp1 and MaSp2 mRNAs have been detected in the tubuliform gland [32]. In black widows tubuliform silk has been shown to contain the spidroin TuSp1, along with Egg Case Protein 1 and 2 (ECP1 and ECP2) [33,34]. Although a comprehensive proteomic analysis of tubuliform silks and the tubuliform gland has yet to be performed using multiple chemical solvents, it will be interesting to determine whether tubuliform silks are assembled with MaSp1 and MaSp1 molecules. If so, this would suggest spiders are capable of complex patterns of silk gene expression in the same abdominal silk-producing gland, creating an opportunity to build a wider range of composite materials that are used in nature. The importance of investigating whether composite spider silk fibers are more common in nature than previously realized by the scientific community has substantial implications, one that potentially extends into synthetic fiber production, which are largely focused on spinning threads from a single recombinant spidroin.

## 4. Experimental Section

### 4.1. Silk Collection

Adult *L. hesperus* females were fed 2–3 weeks prior to silk collection and dissection. MA silk fibers were obtained by using a free-fall or gravity silking methodology. Briefly, MA silk was wrapped around a Y-shaped wooden apparatus during the silking process. Spiders were placed on the Y-apparatus and then dislodged to promote MA silk extrusion. Distance between the two arms of the Y-shaped stick was 6 cm. MA threads were collected by spooling threads onto the Y-apparatus, which involved wrapping dragline silk around the Y-apparatus in a horizontal manner 60 times until a desired length of approximately 4 yards were collected. If spiders broke their dragline silk prior to the targeted length being collected, samples were discarded. Samples were transferred from the Y-apparatus to a sterilized glass hook constructed from Pasteur pipets for subsequent biochemical analysis with different solvents. After collection, samples were immediately stored at −80 °C prior to solvent treatment. Over a 48 h period, 5 replicates of the same length of dragline silk were collected from the same individual.

### 4.2. Gland Dissection

Spiders were anesthetized using CO_2_ gas at 5–10 pounds per square inch for 10 min. Anesthetized spiders were dissected as previously described [35]. Briefly, using micro scissors, the exoskeleton was removed by cutting laterally along both sides of the abdomen until the spinnerets were reached. Exoskeleton segments were peeled back anteriorly using forceps and pinned, exposing fatty tissue. The abdomen was submerged in dissecting buffer solution (0.1 M sodium chloride, 0.015 M sodium citrate, 0.1% diethyl pyrocarbonate). Fat layers were teased away using forceps until silk glands were clearly visible. MA glands, which are found in pairs in one individual, are easily distinguishable by their crescent shaped ampulla with a long convoluted distal tail and a proximal duct (Figure 1B). These structures were teased away carefully and were transferred to a new dish containing dissecting buffer to ensure there were no breaks or punctures. One MA gland was used for proteomic analysis, while the second gland was carefully skinned to obtain pure spinning dope. Both the MA gland and spinning dope were stored at −80 °C until use. If the gland and the spinning dope could not be retrieved from the same individual, the samples were discarded.

### 4.3. Protein Extraction

Five different solvents were used for protein extraction from the silk fibers: 9 M LiBr, 8 M urea, 8 M GdnHCl, 8 M GITC, and 100% HFIP. Reducing agent 1,4-dithiothreitol (DTT) was added to all samples except for HFIP. Samples dissolved in LiBr, GdnHCl, and GITC were placed in 95 °C, and samples containing urea and HFIP were incubated at 25 °C. Fibers were examined at 15-minute intervals to check for complete solubilization. LiBr completely dissolved the fibers within 30 min, while threads placed in urea showed no change after 3 h. GdnHCl treatment dissolved the majority of the fibers after 3 h, but did not fully solubilize. Silk fibers placed into 8 M GITC dissolved immediately after addition of the solvent. HFIP completely dissolved the silk fibers in 30 min. After treatment with HFIP, samples were dried and re-suspended in 8 M GdnHCl supplemented with 10 mM DTT and then heated at 95 °C. Treatment with the other 4 solvents remained at their respective temperatures 3 h. All samples were centrifuged to remove excess material that failed to dissolve. BCA assays were conducted to determine protein concentrations.

One hundred percent HFIP was used for protein extraction of the major ampullate glands. The opaque colored gland and clear, viscous spinning dope for the same individual were dissolved in the same solvent. The gland and spinning dope were solubilized in HFIP overnight at room temperature. After 24 h, both the gland and the spinning dope did not completely dissolve and had small, suspended fragments floating in the solution. Samples were dried and re-suspended in 8 M GdnHCl containing 10 mM DTT then heated at 95 °C. BCA assays were conducted according to the manufacturer’s instructions.

### 4.4. Tryptic Digestion

Proteins from silk fibers and major ampullate glands were dissolved in solvent, alkylated using 15 mM iodoacetamide, diluted with 3 volumes of ammonium bicarbonate (50 mM) and then digested overnight at 37 °C with trypsin (10:1 ratio protein to trypsin). Digested peptides were purified using C18 spin columns according to the manufacturer’s instructions (Thermo Fisher Scientific, Waltham, MA, USA). Purified peptides were dried, re-suspended in water and protein concentrations determined at 280 nm. Using these concentrations, dissolved silk samples were normalized to the same concentration of 0.25 mg/mL and formic acid was added to 0.1%. All samples were stored at −80 °C until analysis.

### 4.5. Chromatography

Each digested silk sample was analyzed three times, each with a different fragmentation method. Prior to each sample injection, the column was washed by injecting water and running CID fragmentation with the same chromatography method that was used in the three subsequent runs. Likewise, after each sample was analyzed a second wash was performed in the same manner. For analysis, 250 ng of each digested sample were sequentially loop injected by a Dionex Ultimate 3000 autosampler connected to an Easy-Spray PepMax^®^ C18 column (75 micron i.d. × 15 cm, 100 A, Thermo Fisher Scientific). Solvents A and B were 0.1% formic acid in water and in acetonitrile, respectively. Solvent B was used at the following concentrations and times: 1%, 3 min, 1%–50%, 30 min, 50%–99%, 12 min, 99%, 5 min, return to 1%, 5 min, 1%, 5 min. Flow rates were held at 300 nL/min. Each run took 1 h; total runtime for all 7 samples was 35 h.

### 4.6. Mass Spectrometry

Mass spectrometry analysis was performed using an Orbitrap Fusion™ Tribrid™ mass spectrometer equipped with an Easy-Spray ion source (Thermo Fisher Scientific) operated in a data dependent manner by Xcalibur 4.0 software (Thermo Fisher Scientific). Full scans were taken by the orbitrap of the analyte with an automatic gain control (AGC) target of 1 million and a maximum inject time of 100 ms using a quadrupole isolation window between *m/z* 300–1500 Da, and resolution setting of 120,000. Monoisotopic peak determination was used, relaxing restrictions when too few peptides were found. Any precursor ion between charges of +2 and +8 surpassing an intensity threshold of 100,000 were subject to fragmentation (quadrupole isolation window: *m/z* 1 Da, AGC target: 100,000, inject time: 200 ms), and were then dynamically excluded for one minute. Three fragmentation methods were used (CID collision 35%, HCD collision 35%, and ETD), one method for each chromatography run, and the fragmentation products were resolved in the ion trap at normal scan rate and an *m/z* range set to automatic. Together, the 21 runs generated 47,824 MS/MS spectra that could be analyzed computationally.

### 4.7. Data Analysis

MS/MS data was analyzed with Proteome Discoverer 2.1 (Thermo Fisher Scientific). An annotated Uniprot protein database for *L. hesperus* was downloaded March 17, 2016, containing all SwissProt and Trembl entries. In addition, a *L. hesperus* transcriptome from was downloaded on 29 July 2016 from NCBI (BioProject PRJNA242358) and converted to an unannotated proteome fasta file using tools from galaxy.org [12]; briefly getORF was used to find open reading frames (ORFs), and then ORFs were converted to proteins using a translate tool, creating an unassembled databases with redundancies. A third custom database was manually made with known protein sequences that were not present in the Uniprot database, namely CRP1 [10] and 17 silk-specific transcripts with scaffold IDs as follow: 433, 213, 118, 355, 21.6, 86, 315, 112, 235, 390, 46, 57, 117, 133, 143, 241, and 330 [9]. Precursor ions with a mass between 550 and 8500 Da were searched against the Uniprot database, using Sequest HT with a precursor mass tolerance of 500 ppm and fragment mass tolerances of 0.6, 0.6 and 1.2 Da for CID, HCD, and ETD, respectively. Two fixed modifications were allowed, carbamidomethylation of cysteine and N-terminal acetylation, as well as the dynamic modification for oxidation of methionine. In addition, phosphorylation of serine, threonine, and tyrosine were allowed as dynamic modifications for analysis of HCD datasets. MS/MS spectra that did not have a peptide match after being searched against the Uniprot database were subsequently searched against the unannotated transcriptome database using the same parameters. Only hits that had 2 or more peptide spectral matches (PSMs) with a false discovery rate (FDR) of 5% were included in graphs (Figure 3, Figure 4, Figure 5 and Figure 6, Figure S2). Later searches of the small custom database, along with the Uniprot database to add sufficient sequences for decoy generation, were performed with the same parameters. As a final comparison, the Venn diagram and heat map (Figure 8 and Figure 9, Figure S3) was generated from all hits (one PSM or more) from the annotated database with a protein FDR of 0.1%.

## 5. Conclusions

In these studies, we demonstrate that using a comprehensive panel of different chemical solvents, followed by in-solution tryptic digestions of the solubilized MA glandular material and dragline silk fibers and nanoLC MS/MS analysis with an Orbitrap Fusion™ Tribrid™ mass spectrometer equipped with three different modes of molecular fragmentation for tryptic peptides can be an effective method to identify components of dragline silk. It also highlights how solvent choice can influence the ability to detect specific proteins in silk fibers. In addition, by analyzing the MA gland, spinning dope, and fibers, it allows investigators to rapidly pinpoint which factors are most highly represented in dragline silk fibers, particularly by focusing on the proteins that are found in all three structures. In closing, these studies provide an exciting set of targets that can be further explored as a fundamental recipe to produce synthetic spider silk with constituents that most closely resemble natural fibers. Further analysis will need to be performed to elucidate whether the identified proteins play a role in fiber mechanics or are extruded with the fibers and serve some other biological function.

## Supplementary Materials

Supplementary materials can be found at www.mdpi.com/1422-0067/17/9/1537/s1.
