# Supplementary material for: Comprehensive Proteomic Analysis of Spider Dragline Silk from Black Widows: A Recipe to Build Synthetic Silk Fibers
Source: Int J Mol Sci. 2016 Sep 13;17(9):1537. doi: 10.3390/ijms17091537 (PMC5037812; doi:10.3390/ijms17091537)
Supplement: Supplementary file 1 [file ijms-17-01537-s001.pdf]

Supplementary Materials: Comprehensive Proteomic Analysis of Spider Dragline Silk from Black Widows: A Recipe to Build Synthetic Silk Fibers

Camille Larracas, Ryan Hekman, Simmone Dyrness, Alisa Arata, Caroline Williams, Taylor Crawford and Craig A. Vierra

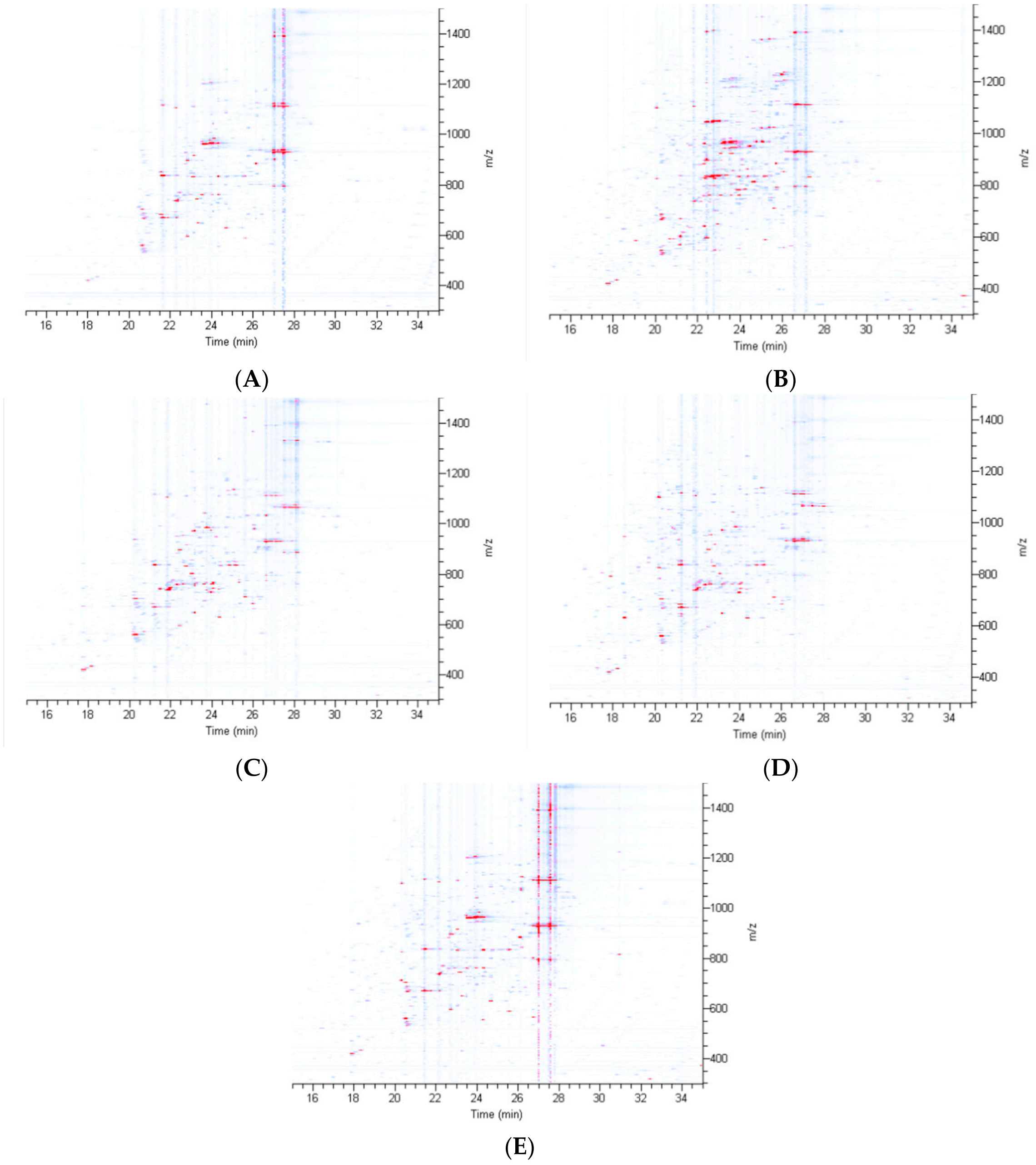

Figure S1. Density map of in-solution tryptic digestion products obtained from dragline silk treated with different chemical solvents. The *X*-axis reveals the retention time (min) for the precursor ions eluted during nano-LC MS analysis, while the *Y*-axis shows the m/z values (z = +2 to +8); (**A**) GdnHCl; (**B**) GITC; (**C**) urea; (**D**) LiBr; (**E**) HFIP.

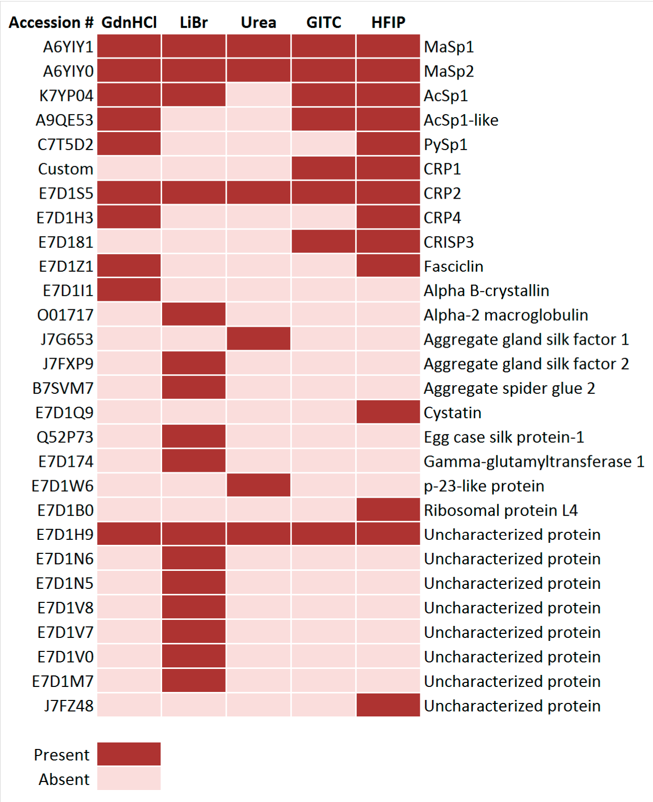

Figure S2. Chart of presence (dark) or absence (light) of proteins that are predicted within dragline silk using all 5 solvents. Accession numbers are shown on the left and names of the proteins on the right.

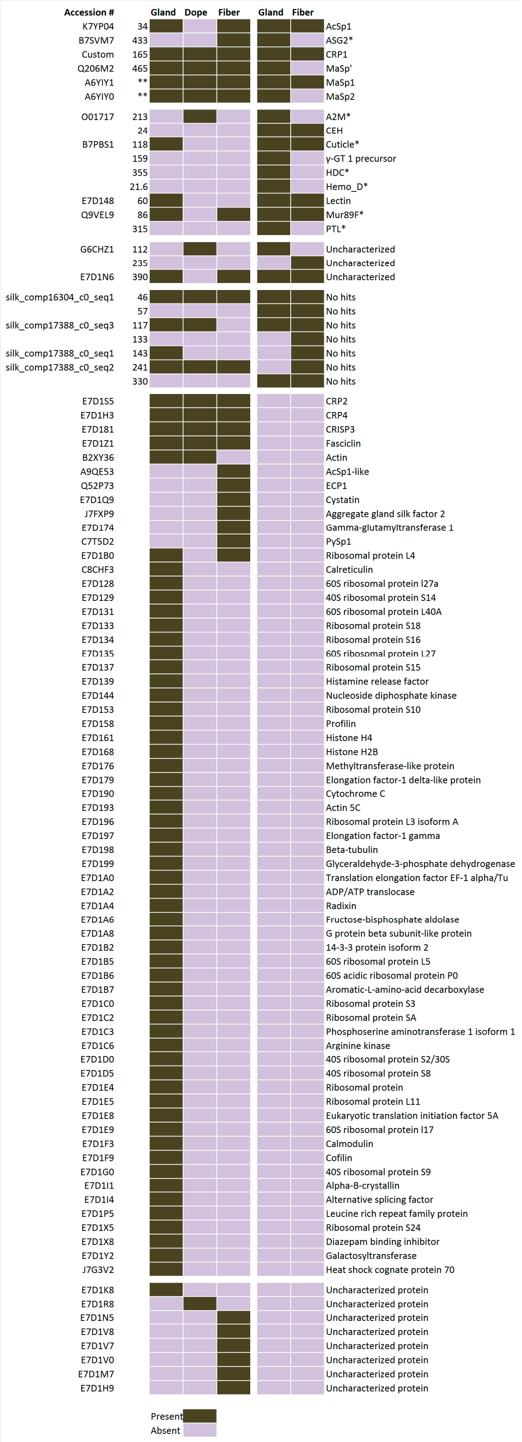

Figure S3. Chart of presence (black) or absence (light purple) of proteins that are predicted within the MA gland, dope and fiber. Two proteomic data sets are available from *L. hesperus* for comparative purposes. The left panel (gland, dope, and fiber) were conducted with an Orbitrap Fusion™ Tribrid™ mass spectrometer with 5 different solvent treatments and 3 modes of peptide ion dissociation (CID, HCD, and ETD), while the right panel (gland, fiber) was conducted using a LTQ Orbitrap Velos™ mass spectrometer and one mode of ion dissociation (CID) [9]. Accession numbers, scaffolding IDs, and SSTs are listed on the left [9]. Custom indicates the sequence was added into the database manually. No hits indicates the sequences do not match anything in the databases. * Indicate some peptide sequences that were derived from SSTs that are uncharacterized in *L. hesperus*, but are thought to be homologous to known proteins in different species. ** Indicate protein types with more than one predicted protein [9].
